# EMA‐Derived Daily Socialization in Younger Older Adults with and without Cognitive Impairment

**DOI:** 10.1002/alz70857_107373

**Published:** 2025-12-26

**Authors:** Marina Kaplan, Sophia L. Holmqvist, Riya Chaturvedi, Tania Giovannetti

**Affiliations:** ^1^ Temple University, Philadelphia, PA, USA

## Abstract

**Background:**

Socialization is known to benefit cognition, but mechanisms remain poorly understood. We explored the relations of EMA‐captured daily socialization and cognitive abilities after accounting for daily activity level and stress.

**Method:**

55 community dwelling older adults (Mean age = 68.6 (SD = 6.99), N, % women = 32, 58.2%, black/African American = 30.9%, NHC = 44, 80 % MCI = 11, 20%) were enrolled in a four‐week study where participants completed a daily 9‐question EMA survey on a smartphone app, and were asked how many hours they spent socializing that day and to rate their daily stress. Daily step count was captured via a Garmin Vivosmart 4 smartwatch which was worn 23 hours per‐day throughout the four weeks. Participants completed a cognitive assessment battery. Multiple linear regressions were used to examine the effects of socialization on cognition. Hierarchical regressions were used to examine whether associations between socialization and cognition remained significant after accounting for activity level and stress.

**Result:**

In separate linear regression models, higher daily socialization was significantly associated with higher cognitive composite (β = .289 p = .027), higher language composite (β = .299 p = .027), but not memory composite (β = .222 p = .103) nor executive function composite (β = .215 p = .114). In hierarchical linear regression models predicting the cognitive composite, daily steps attenuated the relation between socialization and cognitive composite (β = .244, *p* = .057) and daily steps were significant (β = .345, p = .008). Perceived stress was not significantly associated with the cognitive composite. By contrast in hierarchical linear regression models predicting the language composite, neither daily stress nor step count attenuated the relation between socialization and language.

**Conclusion:**

Our results showed higher levels of socialization were specifically associated with better global cognition and better language abilities and that activity has a greater benefit on global cognition than socialization. Thus, socialization may affect various cognitive domains more than others through different mechanisms.

